# Low resting heart rate is associated with violence in late adolescence: a prospective birth cohort study in Brazil

**DOI:** 10.1093/ije/dyv340

**Published:** 2016-01-28

**Authors:** Joseph Murray, Pedro C Hallal, Gregore I Mielke, Adrian Raine, Fernando C Wehrmeister, Luciana Anselmi, Fernando C Barros

**Affiliations:** ^1^ Department of Psychiatry, University of Cambridge, Cambridge, UK,; ^2^ Postgraduate Program in Epidemiology, Universidade Federal de Pelotas, Pelotas, Brazil,; ^3^ Departments of Criminology, Psychiatry, and Psychology, University of Pennsylvania, Philadelphia, PN, USA and; ^4^ Postgraduate Programme in Health and Behavior, Universidade Católica de Pelotas, Pelotas, Brazil

**Keywords:** Heart rate, violence, crime, cohort study

## Abstract

**Background** : Youth violence is a major global public health problem. Three UK and Swedish studies suggest that low resting heart rate predicts male youth violence, but this has not been tested in other social settings nor for females.

**Methods** : A prospective, population-based birth cohort study was conducted in Pelotas, Brazil. Heart rate was measured using a wrist monitor at ages 11, 15 and 18 years. Violent crime and non-violent crime were measured at age 18 in self-reports and official records ( *N* = 3618). Confounding variables were assessed in the perinatal period and at age 11, in interviews with mothers and children. Logistic regression was used to estimate associations between quartiles of heart rate at each age, and violent and non-violent crime at age 18, separately for males and females.

**Results** : Lower resting heart rate was a robust correlate of violent and non-violent crime for males. Comparing males in the lowest and top quartiles of heart rate at age 15 years, adjusted odds ratios were 1.9 for violent crime [95% confidence interval (CI) 1.4–2.7] and 1.7 for non-violent crime (95% CI 1.1–2.6). For females, crime outcomes were associated only with low resting heart rate at age 18. Associations were generally linear across the four heart rate quartiles. There was no evidence that associations differed according to socioeconomic status at age 15.

**Conclusions** : Low resting heart rate predicted violent and non-violent crime for males, and was cross-sectionally associated with crime for females. Biological factors may contribute to individual propensity to commit crime, even in a middle-income setting with high rates of violence.

Key Messages

- Violent and non-violent crime are influenced by biological, psychological and social factors, but most criminological research has focused on social influences.
- Three previous studies in the UK and Sweden suggest that violent males have low resting heart rates.
- The highest rates of serious violence occur in Latin America and the Caribbean, but prospective studies of violence are almost non-existent in those regions.
- We conducted a large, prospective, population-based study in a Brazilian city.
- Low resting heart rate predicted violent and non-violent crime through adolescence for males, and was concurrently associated with crime at age 18 for females.

## Introduction

Youth violence is a major global public health problem. In 2010, 25.5 million healthy life-years were lost worldwide due to injuries resulting from interpersonal violence. ^1^ Mortality from violence is exceptionally high in Latin America and the Caribbean where it was the second leading cause of years of life lost, after heart disease, in 2013. ^2^ In Brazil, homicide rates rose rapidly between 1980 to 2010, ^3^ and became the leading cause of death among people aged 15–29 years. ^4^ Non-lethal violence is also a considerable problem inBrazil. ^3^^,^^5^ Violence is influenced by numerous individual, relationship, community and society-wide processes. ^6^^,^^7^ However, most research has focused on social influences, and nearly all longitudinal studies have been conducted in high-income countries in North America, Europe and Australasia. ^6^^,^^8^^,^^9^

An intriguing suggestion concerning biological influences is that individuals with low resting heart rates have increased risk for violence. Heart rate is affected by the sympathetic and parasympathetic branches of the autonomic nervous system, which in turn is influenced by the central autonomic network comprising cortical, limbic and midbrain structures. ^10^ Low resting heart rate might correlate with violence because it indicates fearlessness or a state of low autonomic arousal, which causes sensation-seeking behaviour including violence. ^11–13^

Four previous studies have examined the association between heart rate and violence, all based on male samples in high-income countries. The first included 1831 males in the 1946 British National Survey of Health and Development. ^14^ At age 11, average heart rates were lower among males with police records for violence or any crime aged between 8–21 years, compared with non-offenders, but no adjustment was made for confounding factors. A second UK study included 411 working-class London males born in 1953 (the Cambridge Study in Delinquent Development), among whom low resting heart rate at age 18 years was associated with self-reported violence at the same age and conviction for total and violent offences between ages 10 and 50, adjusting for numerous individual and family covariates. ^15^^,^^16^ A third study in the USA included 40 men who had previously committed partner assault, among whom heart rate reactivity was not associated with non-partner violence. ^17^ Most recently, in a register-based study of 710 000 Swedish men born in 1958–91, low resting heart rate at age 18 years predicted official records of adult violence (mean follow-up 18 years) adjusting for physical, cognitive, psychiatric and socioeconomic covariates. ^18^ Low resting heart rate was more strongly associated with violent crime (particularly severe violence) than non-violent crime among Swedish men. ^18^ In a meta-analysis of the UK and US studies, Portnoy and Farrington ^12^ found an average association between heart rate and violence of d = −.35, a stronger effect size than for any other form of antisocial behaviour in their review—including conduct disorders, aggression, behaviour problems, general offending and psychopathy.

It would be highly desirable to conduct new tests of the heart rate-violence relationship in recent samples, also including females and in other social settings. The current study tests whether low heart rate is associated with both violent and non-violent crime, among females as well as males, in a large Brazilian birth cohort.

## Methods

### Participants

**Figure 1.**
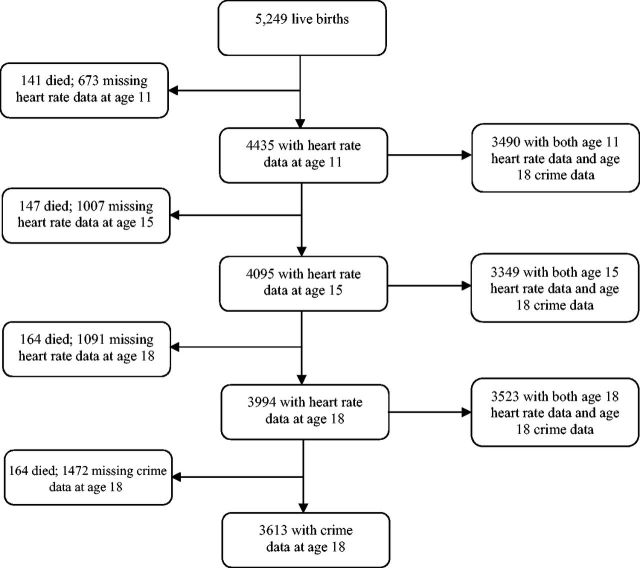
Flow chart of data collected on heart rate and crime in the 1993 Pelotas Birth Cohort Study.

We used data from the 1993 Pelotas (Brazil) Birth Cohort Study. Detailed description of the study methods and sample is available elsewhere. ^19^^,^^20^ Briefly, all births occurring in the five maternity clinics in the city of Pelotas, Southern Brazil, were monitored in 1993 (99% of births in Pelotas occurred in hospital). For the 5265 children born alive, only 16 mothers could not be interviewed or refused to participate. The 5249 newborns whose mothers lived in the urban area were included in the cohort. At ages 11, 15 and 18 years, 87.5%, 85.7% and 81.3% of the original cohort members were followed, respectively. Figure 1 shows the availability of data at each follow-up. Each assessment was approved by the Research Ethics Committee of the Federal University of Pelotas School of Medicine. Participants provided written informed consent at each stage of the study.

### Measures

#### Resting heart rate

Resting heart rate at ages 11, 15 and 18 years was measured using a digital monitor (Omron brand, model 711-AC; Beijing, China). The monitor was attached to the wrist of the participant while in a sitting position. Two measures were taken, first at the beginning of the assessment session (after resting for 10 min) and the second at the end of the assessment procedures. The mean of the two measurements was calculated and used in the analyses. At each age (11, 15 and 18 years), quartiles of resting heart rate were calculated and used as predictor variables in the analyses.

#### Violent and non-violent crime

There are two main methods for measuring criminal behaviour: the first based on self-reports and the second on searches of criminal records. Criminal records have the advantage of including more serious offences, and an advantage of self-reports is that they include crimes that do not come to the attention of criminal justice agencies. We combined data from self-reports and criminal records to identify participants who perpetrated at least one crime in late adolescence. At age 18, participants completed a confidential self-report questionnaire asking about crimes committed in the previous 12 months. ^5^ Due to a printing error, the first 325 questionnaires (8% of 4106 participants) were not useable. The current analyses include the vast majority of participants ( *N* = 3618) with complete crime data. Criminal records were searched in police, court and juvenile justice institutions in Pelotas city and the state of Rio Grande do Sul. Participants who either self-reported at least one crime at age 18 or who had a criminal record between ages 16 and 18 were classified as having committed a crime. Violent crimes referred to assault, robbery and carrying and using weapons in self-reports, and also to homicide, kidnapping, sexual crimes, serious personal threats and other rare violent acts in criminal records. All other crimes were classified as non-violent crimes, except traffic offences which were excluded from analyses.

#### Confounding variables

The following variables were measured in the perinatal period in interviews with mothers: unplanned pregnancy (yes/no), mother smoked in pregnancy (yes/no), maternal alcohol use in pregnancy (yes/no), maternal age (years), number of siblings, maternal education (years of schooling) and family income (minimum wages per month). All were previously found to be associated with violence at age 18 years for men or women in this sample. ^21^ Self-reported skin colour was measured and categorized as white, black, mullato/brown, yellow and indigenous. The following participant characteristics were measured at age 11 years: smoking (yes/no), drinking (yes/no), physical activity (min per week in leisure time physical activity and active transportation to/from school), height (centimetres), weight (kilograms), and blood pressure (mmHg continuous). Maternal mental health was also measured when participants were aged 11, using the Self Report Questionnaire (SRQ), previously validated in a Brazilian sample of 485 subjects. ^22^ The continuous SRQ score from 0 to 20 was used in the analyses.

### Statistical analyses

Odds ratios and 95% confidence intervals were calculated for the associations between quartiles of resting heart rate at each age (11, 15, 18 years) and violent and non-violent crime, using logistic regression. Multivariate logistic regression was used to adjust for confounding variables. *P* -values were calculated using a Wald test for linear trend or for heterogeneity when a deviation of linearity was observed. All analyses were stratified by sex. The decision to do this was taken a priori because of sex differences in crime rates and heart rates and because all previous studies included only males; formal tests of interaction between sex and heart rate in predicting violent and non-violent crime were also performed, finding an interaction for violence at 15 years ( *P* = 0.029). In sensitivity analyses, results were re-estimated using the first and second measures of heart rate at age 18 separately (rather than the average of the two measures). Also, analyses were re-run estimating missing data using multiple imputation. Fifty data sets (each with 2603 males and 2645 females) were created using the *mi impute chained* command in STATA, in which all missing data (except sex, which was missing for one participant) were predicted in logistic and linear regression models.

## Results

The prevalence of violent crime at age 18 was 26.6% among men and 11.3% among women ( *P* < 0.001). Equivalent figures for non-violent crime were 14.8% and 5.8%, respectively ( *P* < 0.001). Among those who committed violence ( *N* = 679), less than half (44%) also committed a non-violent crime; by contrast, among those who committed non-violent crime ( *N* = 367) the vast majority (82%) also committed violence.

Mean heart rate at 11 years was 76.5 beats per minute (bpm) among boys and 80.2 bpm among girls. Equivalent figures at age 15 years were 79.4 bpm and 84.1 bpm, respectively. At age 18, mean heart rate was 69.9 bpm among men and 77.9 bpm among women.

Table 1 displays the quartiles of heart rate at each age, which were created by splitting the total cohort (males and females together) into four equally-sized groups, with the bottom quartile having the lowest heart rate and the top quartile having the highest heart rate. The mean heart rate in the bottom quartile at age 11 was 65 bpm for males and 66 bpm for females, whereas the top quartile had mean values of 92 bpm for males and 93 bpm for females. Values were similar at age 15 years. At age 18 years, the mean of the bottom quartile for men and women was 59 bpm and 61 bpm, respectively. For the fourth quartile, mean values were 90 bpm for both men and women.

**Table 1.** Description of quartiles of heart rate at ages 11, 15 and 18 years.

		Men			Women	
Age	*N*	Mean (SD)	Median (25–75)	*N*	Mean (SD)	Median (25–75)
11 years						
4 (highest)	430	92.4 (6.6)	90.5 (88.0–95.0)	652	93.3 (7.5)	91.5 (88.0–96.5)
3	480	81.4 (2.0)	81.3 (79.5–83.0)	581	81.6 (2.1)	81.5 (79.5–83.5)
2	537	74.8 (2.0)	75.0 (73.0–76.5)	573	74.9 (2.0)	75.0 (73.0–76.5)
1 (lowest)	732	65.3 (4.4)	66.0 (62.5–69.0)	450	66.3 (4.2)	67.0 (64.5–69.5)
15 years						
4 (highest)	370	98.1 (7.4)	96.0 (92.5–102)	643	98.9 (7.6)	96.5 (93.0–103)
3	446	85.0 (2.5)	85.0 (82.5–87.0)	524	85.1 (2.3)	85.0 (83.0–87.0)
2	535	77.3 (2.3)	77.5 (75.5–79.5)	517	77.5 (2.3)	77.5 (75.5–79.5)
1 (lowest)	653	66.6 (5.1)	67.5 (63.5–70.5)	407	67.9 (4.6)	69.0 (65.5–71.5)
18 years						
4 (highest)	320	90.4 (7.9)	88.5 (84.0–94.5)	669	90.4 (8.3)	88.0 (84.5–94.0)
3	346	76.9 (2.3)	77.0 (75.0–79.0)	594	77.2 (2.3)	77.0 (75.0–79.0)
2	506	69.4 (2.1)	69.3 (67.5–71.0)	525	69.8 (2.1)	70.0 (68.0–71.5)
1 (lowest)	814	59.2 (4.7)	60.5 (56.0–63.0)	220	61.3 (3.4)	62.3 (59.0–64.0)

**Figure 2:**
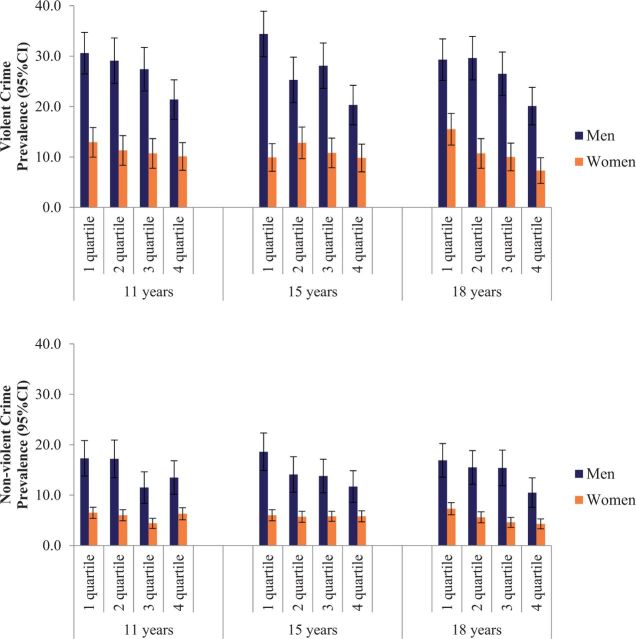
Prevalence (%) of a) violent crime and b) non-violent crime at 18 years, according quartiles of heart rate at 11 years, 15 years and 18 years.

Figure 2 . displays the unadjusted associations between heart rate and (a) violent and (b) non-violent crime. Among men, lower heart rate was associated with greater risk for crime in an almost linear pattern, whereas clear associations were not observed for females. For example, the prevalence of violent crime was 34% among men in the first quartile of heart rate at 15 years, whereas it was 20% among those in the fourth quartile. Equivalent figures for non-violent crime were 19% and 12%, respectively.

Table 2 presents unadjusted and adjusted odds ratios for violent crime. Males in the bottom quartile of heart rate at 11 years had 62% higher odds of crime compared with those in the top quartile. Males in the bottom quartile at 15 years had 106% higher odds of violent crime than those in the top quartile. At age 18, the cross-sectional association between heart rate and violent crime among men was odds ratio (OR) = 1.65. Adjustment for confounders did not considerably change the magnitude of the associations. For women, heart rate at ages 11 and 15 years did not predict violent crime either in crude or in adjusted analyses. Cross-sectional associations, however, were in the expected direction. Women in the bottom quartile of heart rate presented unadjusted odds 115% higher than those in the top quartile. In the adjusted analyses, the increased odds were 111%.

**Table 2.** Odds ratio (OR) for violent crime at age 18 according to heart rate quartiles at ages 11, 15 and 18 years in the 1993 Pelotas Birth Cohort

	Men ^a^				Women ^a^			
	Crude		Adjusted		Crude		Adjusted	
	OR (95% CI)	*P* -value ^b^	OR (95% CI)	*P* -value ^b^	OR (95% CI)	*P* -value ^b^	OR (95% CI)	*P* -value ^b^
Heart at 11 years		0.002		0.028		0.170		0.045
4 (highest)	1.00		1.00		1.00		1.00	
3	1.38 (1.00–1.91)		1.35 (0.96–1.92)		1.07 (0.69–1.65)		1.07 (0.66–1.74)	
2	1.50 (1.09–2.07)		1.41 (1.07–2.13)		1.13 (0.74–1.72)		1.20 (0.75–1.93)	
1 (lowest)	1.62 (1.19–2.20)		1.46 (1.01–1.65)		1.32 (0.88–1.99)		1.55 (0.98–2.44)	
Heart at 15 years		<0.001		0.001		0.477 ^c^		0.339 ^c^
4 (highest)	1.00		1.00		1.00		1.00	
3	1.54 (1.11–2.13)		1.49 (1.05–2.11)		1.11(0.71–1.72)		1.13 (0.70–1.85)	
2	1.33 (0.95–1.86)		1.19 (0.83–1.71)		1.34 (0.87–2.06)		1.41 (0.88–2.28)	
1 (lowest)	2.06 (1.51–2.82)		1.90 (1.36–2.67)		1.01 (0.65–1.57)		0.96 (0.59–1.60)	
Heart at 18 years		0.001		0.038		0.001		0.005
4 (highest)	1.00		1.00		1.00		1.00	
3	1.43 (1.04–1.98)		1.41 (0.99–2.00)		1.40 (0.86–2.26)		1.52 (0.89–2.61)	
2	1.67 (1.22–2.29)		1.49 (1.06–2.11)		1.52 (0.95–2.44)		1.59 (0.93–2.71)	
1 (lowest)	1.65 (1.21–2.24)		1.47 (1.04–2.06)		2.15 (1.37–3.36)		2.11 (1.27–3.50)	

Covariates included in adjusted model: unplanned pregnancy, mother smoked in pregnancy, maternal alcohol use in pregnancy, maternal age, number of siblings, maternal education and family income in perinatal period; child skin colour, smoking, drinking, physical activity, height, weight, blood pressure; mother’s mental health at child age 11 years.

^a^ *N* for men between 1753 and 1462; *N* for women between 1805 and 1566.

^b^ Wald test for linear trend.

^c^ Wald test for heterogeneity.

Table 3 shows the crude and adjusted associations between heart rate and non-violent crime. For males, heart rate at age 11 predicted 35% higher odds of non-violent crime in the unadjusted analysis and 21% in adjusted analyses. For heart rates at ages 15 and 18, males in the bottom quartile of heart rate had 68% and 60% higher odds of non-violent crime, respectively, in adjusted models. Similar to the findings for violent crime, heart rate at ages 11 and 15 years did not predict non-violent crime among women. However, cross-sectionally women in the bottom quartile of heart rate at age 18 had a 76% increased odds for non-violent crime in the unadjusted analysis and 232% in the fully adjusted model.

**Table 3.** Odds ratio (OR) for non-violent crime at age 18 according to heart rate quartiles at ages 11, 15 and 18 years in the 1993 Pelotas Birth Cohort

	Men ^a^				Women ^a^			
	Crude		Adjusted		Crude		Adjusted	
	OR (95% CI)	*P* -value ^b^	OR (95% CI)	*P* -value ^b^	OR (95% CI)	*P* -value ^b^	OR (95% CI)	*P* -value ^b^
Heart at 11 years		0.026		0.095 ^c^		0.565 ^c^		0.127
4 (highest)	1.00		1.00		1.00		1.00	
3	0.83 (0.55–1.26)		0.75 (0.47–1.17)		0.69 (0.38–1.26)		0.81 (0.42–1.58)	
2	1.33 (0.91–1.95)		1.22 (0.80–1.85)		0.94 (0.55–1.63)		1.07 (0.58–2.00)	
1 (lowest)	1.35 (0.93–1.95)		1.21 (0.81–1.81)		1.03 (0.61–1.75)		1.45 (0.80–2.63)	
Heart at 15 years		0.007		0.022		0.757		0.328
4 (highest)	1.00		1.00		1.00		1.00	
3	1.20 (0.79–1.83)		1.26 (0.81–1.98)		0.87 (0.49–1.57)		0.89 (0.45–1.73)	
2	1.23 (0.81–1.87)		1.22 (0.77–1.92)		0.98 (0.55–1.76)		1.13 (0.59–2.18)	
1 (lowest)	1.72 (1.17–2.54)		1.68 (1.10–2.57)		1.05 (0.60–1.84)		1.26 (0.68–2.34)	
Heart at 18 years		0.010		0.092		0.038		0.012
4 (highest)	1.00		1.00		1.00		1.00	
3	1.55 (1.03–2.34)		1.48 (0.94–2.33)		1.09 (0.57–2.10)		1.42 (0.66–3.03)	
2	1.56 (1.04–2.33)		1.37 (0.87–2.16)		1.33 (0.72–2.48)		1.75 (0.84–3.63)	
1 (lowest)	1.74 (1.17–2.57)		1.60 (1.03–2.47)		1.76 (0.98–3.17)		2.32 (1.15–4.67)	

Covariates included in adjusted model: unplanned pregnancy, mother smoked in pregnancy, maternal alcohol use in pregnancy, maternal age, number of siblings, maternal education and family income in perinatal period; child skin colour, smoking, drinking, physical activity, height, weight, blood pressure, mother’s mental health at child age 11 years.

^a^ *N* for men between 1753 and 1462; *N* for women between 1805 and 1566.

^b^ Wald test for linear trend.

^c^ Wald test for heterogeneity.

We tested for possible interactions between heart rate and family income in determining violent and non-violent crime. Out of 12 interaction tests (3 ages of heart rate data * 2 types of crime * 2 sexes), none showed strong evidence of interaction: 10 of the *P* -values were above 0.40, and all were above 0.10.

### 

#### Sensitivity analyses

Table S1 (available as Supplementary data at *IJE* online) shows that participants with and without crime data at age 18 were similar on all variables except heart rate at age 18, and height, weight and blood pressure at age 11. Supplementary Data and Supplementary Data (available as Supplementary data at *IJE* online) show that, using multiple imputation for missing data, results were very similar compared with results based on complete cases in the main analyses. Supplementary Data (available as Supplementary data at *IJE* online) shows that, using the first and second measures of heart rate at age 18 separately, associations with violent and non-violent crime were similar to results from the main analyses based on the mean heart rate.

## Discussion

The key finding of this study is that lower heart rate was a robust predictor of male violent and non-violent crime. Although only cross-sectional associations were found for females, prospective and concurrent associations were observed for males after adjusting for a range of confounding variables. The main strengths of the study were: (i) the use of a large prospective community cohort; (ii) the repeated measures of heart rate; (iii) the multiple measures of crime; (iv) the inclusion of females as well as males; and (v) the wide range of confounding variables included. To our knowledge, this is the first test of the association in a middle-income country with a high rate of serious violence.

Our findings are consistent with the results from three UK and Swedish studies of men. ^14^^,^^15^^,^^18^ Thus, four studies now provide strong evidence for an association between low resting heart rate and violence among males, across different social settings. The association with non-violent crime in the current study is also consistent with studies of other forms of antisocial behaviour, linking low heart rate with childhood aggressive and non-aggressive conduct problems, adult psychopathy and non-violent crime. ^12^^,^^18^^,^^23–25^ Interestingly, low resting heart rate seems to specifically affect antisocial behaviour: no other psychiatric condition other than conduct disorder has been linked with low heart rate. ^11^ Other psychiatric conditions, including alcoholism, depression, schizophrenia and anxiety disorder, have if anything been linked to higher (not lower) resting heart rate, and the same is true of non-psychiatric conditions such as diabetes. ^26^

There are two main theories about why low resting heart rate might have this specific association with antisocial behaviour. ^11^ The first theory suggests that low resting heart rate reflects low autonomic arousal which is an unpleasant physiological state causing stimulating-seeking behaviour, including antisocial behaviour. ^27^^–^^28^ For example in a Dutch longitudinal study, ^29^ the association between boys’ low heart rate and rule-breaking behaviour was mediated by sensation seeking, and the same was true for aggression in a US sample. ^30^ An important developmental precursor of sensation seeking is behavioural inhibition in childhood, characterized by particular amygdala responses to novelty; thus a disinhibited temperament in childhood may underlie the associations observed in this study. ^31^

A second theory of the association between heart rate and antisocial behaviour focuses on fearlessness. According to this theory, committing antisocial and violent acts requires a degree of fearlessness which is indicated by low levels of arousal during mildly stressful psychophysiological tests. Children who lack fear are less likely to be responsive to socializing punishments, which may in turn contribute to poor fear conditioning and lack of conscience development. ^13^ However, fearlessness did not mediate the relationship between low resting heart rate and aggressive or non-aggressive antisocial behaviour in a US study. ^30^ Raine ^11^ reviewed these and other possible links between low resting heart rate and antisocial behaviour, and concluded that combined effects of stimulation-seeking, fearlessness and reduced noradrenergic functioning probably provide the best explanation, but more research is required.

Associations between heart rate and violent and non-violent crime were generally linear in the current study. Although similar mechanisms may operate at different levels of heart rate in a graded fashion, it is also possible that having a higher heart rate has specific ‘buffering’ effects against the influence of other risk factors. ^32^^,^^33^ In two UK studies, there were interactions between heart rate and social risk factors, including having a large family ^15^ or being separated from a parent. ^14^ Among high-heart-rate individuals, social risk factors had weaker effects on violence compared with low-heart-rate individuals. However, in the current study there was no evidence for an interaction between heart rate, family income level and probability of violent or non-violent crime.

This is the first study to investigate the association between heart rate and violence for females, and was unusual in having three measures of heart rate across adolescence. Females had higher heart rates than males at all ages, consistent with other evidence. ^34^^,^^35^ For females, violent and non-violent crime were associated with lower heart rate at age 18, but not with heart rate at ages 11 or 15. For males, associations were found with lower heart rate at all ages, consistent with previous evidence. ^14^ Why did heart rate predict crime across adolescence for males but not females? One possible explanation is that heart rate was less stable through time for females than for males. For example, the correlation between heart rate at ages 15 and 18 years was 0.45 for males and 0.37 for females. A second possible explanation is that female criminal behaviour is less persistent than male criminal behaviour, and low resting heart rate is more strongly related to persistent antisocial behaviour. ^36^ A third possibility is that, because females have lower crime rates than males, the null findings for females might reflect floor effects. However, other studies of less serious behaviour problems, such as rule-breaking and aggression, have also found weaker, or non-existent effects for females. ^12^^,^^29^

One limitation of the current study was that, although numerous confounding variables were included in the analyses, we were unable to test causal mechanisms. Given the intergenerational continuity in crime ^37^ and heart rate, ^38^ there might be residual confounding in the current study, because measures of parental antisocial behaviour and heart rate were not included. The current study included 3618 participants with valid data at age 18. It is possible that the exclusion of other cases because of missing data influenced the findings, although results based on multiple imputation of missing data were very similar to those based on complete cases. Because of the high rate of violence in this sample, we cannot be sure if low resting heart rate predicted non-violent crime independently of violence—there were too few participants who committed non-violent crime only to analyse them as a separate category. However, in a previous Swedish study with a lower rate of violence, low resting heart rate was associated with non-violent crime even after excluding participants with records of violence. ^18^ It should also be considered that the current study was conducted in one Brazilian city, and results should not be generalized to the rest of the country. Pelotas is a relatively poor city in a relatively rich southern state of Brazil. When crime data were collected for this study in 2011, there were 18.9 homicides in Pelotas per 100 000 population, lower than the national rate of 27.1 but considerably higher than in England and Wales (1.3) and Sweden (0.9) ^39^ where previous studies of heart rate and violence have been conducted.

Future studies should include measures of possible intervening physiological and psychological variables, and employ other designs to strengthen causal inference. One potentially useful direction for future research would be to use a Mendelian randomization design to improve causal inference. ^40^ If genetic variants that influence heart rate were used as instrumental variables, stronger causal conclusions could be drawn about the causal effects of heart rate on antisocial behaviour. If causal effects were identified, the specific cardiovascular processes (e.g. sympathetic versus parasympathetic) active in shaping the relationship between low heart rate and crime should be investigated. Also, replication studies are needed for females, which should consider the stability of both heart rate and crime as possible explanations for why heart rate is less predictive for females than males across adolescence.

It is a striking conclusion that an individual-level biological characteristic, such as heart rate, is associated with crime in a Brazilian sample, given the high levels of serious violence in Brazil. One might speculate that individual-level factors would be irrelevant in this social context, because of major socio-cultural drivers of crime and violence, including poverty, inequality, gangs, drug trafficking and corrupt and under-resourced criminal justice systems. However, the current study demonstrates that, even in this setting, a fully integrated biopsychosocial understanding of violence is required.

## Funding

The 1993 Pelotas (Brazil) Birth Cohort Study is currently supported by the Wellcome Trust through the programme entitled Major Awards for Latin America on Health Consequences of Population Change. The European Union, National Support Program for Centers of Excellence (PRONEX), the Brazilian National Research Council (CNPq), the Foundation for Research Support of the State of Rio Grande do Sul (FAPERGS) and the Brazilian Ministry of Health supported previous phases of the study. J.M. is supported by the Wellcome Trust [grant number 089963/Z/09/Z]. P.H. is supported by the Wellcome Trust through a New Investigator Award.

**Conflict of interest:** The authors declare no conflicts of interest.

## Supplementary Material

Supplementary Data
