# Supplementary material for: Low resting heart rate is associated with violence in late adolescence: a prospective birth cohort study in Brazil
Source: Int J Epidemiol. 2016 Jan 28;45(2):491–500. doi: 10.1093/ije/dyv340 (PMC4864875; doi:10.1093/ije/dyv340)
Supplement: Supplementary Data [file dyv340_supplementary_data.zip › ije-2015-05-0657-File002.docx]

Table S1: Study measures by availability of crime data at age 18

|  | With crime data | Missing crime data |
| --- | --- | --- |
|  | N = 3,618 | N = 1,631 |
| **Heart rate measures** |  | |
| Age 11 (bpm) | *p*=0.512 | |
| Mean (SD) | 78.5 (11.1) | 78.2 (10.5) |
| Age 15 (bpm) | *p*=0.336 | |
| Mean (SD) | 81.9 (12.6) | 81.4 (12.1) |
| Age 18 (bpm) | *p*<0.001 | |
| Mean (SD) | 73.6 (12.2) | 76.2 (13.0) |
| **Perinatal measures** |  | |
| Unplanned pregnancy | *p*=0.571 | |
| Yes | 55.9% | 57.0% |
| Mother smoked in pregnancy | *p*=0.233 | |
| Yes | 32.9% | 34.5% |
| Mother used alcohol in pregnancy | *p*=0.580 | |
| Yes | 5.0% | 5.3% |
| Maternal age (years) | *p*=0.444 | |
| Mean (SD) | 26.0 (6.4) | 25.9 (6.4) |
| Number of pregnancies | *p*=0.123 | |
| Mean (SD) | 2.5 (1.7) | 2.5 (1.8) |
| Maternal education (years) | *p*=0.168 | |
| Mean (SD) | 6.8 (3.5) | 6.6 (3.7) |
| Family income (minimum wages) | *p*=0.827 | |
| Mean (SD) | 4.3 (5.9) | 4.3 (5.8) |
| **Age 11 measures** |  | |
| Participant smoking | *p*=0.117 | |
| Yes | 3.5% | 4.6% |
| Participant drinking alcohol | *p*=0.177 | |
| Yes | 17.1% | 19.0% |
| Participant physical activity (mins/pw) | *p*=0.963 | |
| Mean (SD) | 414 (760) | 416 (780) |
| Participant height (cm) | *p*=0.020 | |
| Mean (SD) | 1.46 (0.08) | 1.46 (0.08) |
| Participant weight (kg) | *p*=0.051 | |
| Mean (SD) | 39.8 (10.2) | 40.5 (11.0) |
| Participant systolic blood pressure | *p*=0.001 | |
| Mean (SD) | 101.5 (12.3) | 103.0 (12.2) |
| Participant diastolic blood pressure | *p*=0.018 | |
| Mean (SD) | 63.2 (9.9) | 64.0 (9.7) |
| Maternal mental health | *p*=0.359 | |
| Mean (SD) | 5.7 (4.5) | 5.8 (4.5) |

Notes. Column percents.

Table S2. Multiple imputation results: Odds Ratio (OR) for violent crime at age 18 according to heart rate quartiles at ages 11, 15, and 18 years in the 1993 Pelotas Birth Cohort.

|  | | **Men**^a^ | | | | | | | |  | | **Women**^a^ | | | | | | | | | |  |  |
| --- | --- | --- | --- | --- | --- | --- | --- | --- | --- | --- | --- | --- | --- | --- | --- | --- | --- | --- | --- | --- | --- | --- | --- |
|  | | Crude | | | |  | | Adjusted | |  | | Crude | | | |  | | Adjusted | | | |  |  |
|  | | OR (95% CI) | | p value^b^ | |  | | OR (95% CI) | p value^b^ |  | | OR (95% CI) | | p value^b^ | |  | | OR (95% CI) | | p value^b^ | |  |  |
| Violent crime | | | |  | |  | |  | |  | |  | |  | |  | |  | |  | |  | |
| Heart at 11 years | |  | | 0.004 | |  | |  | | 0.024 | |  | |  | | 0.076 | |  | |  | | 0.192 | |
| 4 (highest) | | 1.00 | |  | |  | | 1.00 | |  | |  | | 1.00 | |  | |  | | 1.00 | |  | |
| 3 | | 1.38 (1.00-1.90) | |  | |  | | 1.33 (0.96-1.86) | |  | |  | | 1.15 (0.75-1.76) | |  | |  | | 1.05 (0.68-1.62) | |  | |
| 2 | | 1.45 (1.04-2.01) | |  | |  | | 1.40 (0.99-1.96) | |  | |  | | 1.22 (0.80-1.84) | |  | |  | | 1.14 (0.75-1.74) | |  | |
| 1 (lowest) | | 1.59 (1.16-2.17) | |  | |  | | 1.47 (1.06-2.02) | |  | |  | | 1.42 (0.96-2.10) | |  | |  | | 1.29 (0.86-1.94) | |  | |
| Heart at 15 years | |  | | <0.001 | |  | |  | | 0.003 | |  | |  | | 0.318 | |  | |  | | 0.659 | |
| 4 (highest) | | 1.00 | |  | |  | | 1.00 | |  | |  | | 1.00 | |  | |  | | 1.00 | |  | |
| 3 | | 1.47 (1.07-2.03) | |  | |  | | 1.43 (1.03-1.97) | |  | |  | | 1.19 (0.78-1.89) | |  | |  | | 1.15 (0.74-1.79) | |  | |
| 2 | | 1.40 (1.01-1.95) | |  | |  | | 1.29 (0.92-1.81) | |  | |  | | 1.34 (0.89-2.01) | |  | |  | | 1.30 (0.86-1.99) | |  | |
| 1 (lowest) | | 1.88 (1.36-2.58) | |  | |  | | 1.75 (1.27-2.41) | |  | |  | | 1.22 (0.79-1.87) | |  | |  | | 1.08 (0.70-1.67) | |  | |
| Heart at 18 years | |  | | 0.001 | |  | |  | | 0.012 | |  | |  | | 0.001 | |  | |  | | 0.012 | |
| 4 (highest) | | 1.00 | |  | |  | | 1.00 | |  | |  | | 1.00 | |  | |  | | 1.00 | |  | |
| 3 | | 1.42 (1.05-1.92) | |  | |  | | 1.32 (0.97-1.81) | |  | |  | | 1.37 (0.86-2.18) | |  | |  | | 1.31 (0.82-2.10) | |  | |
| 2 | | 1.66 (1.24-2.24) | |  | |  | | 1.52 (1.12-2.06) | |  | |  | | 1.50 (0.96-2.36) | |  | |  | | 1.37 (0.87-2.16) | |  | |
| 1 (lowest) | | 1.69 (1.27-2.26) | |  | |  | | 1.46 (1.08-1.99) | |  | |  | | 1.97 (1.29-3.01) | |  | |  | | 1.72 (1.12-2.63) | |  | |

^a^ 2603 for men; 2645 for women

^b^ Wald test for linear trend

* Wald test for heterogeneity

Covariates included in adjusted model: unplanned pregnancy, mother smoked in pregnancy, maternal alcohol use in pregnancy, maternal age, number of siblings, maternal education, and family income in perinatal period; child skin colour, smoking, drinking, physical activity, height, weight, blood pressure, maternal mental health at 11 years.

Table S3. Multiple imputation results: Odds Ratio (OR) for non-violent crime at age 18 according to heart rate quartiles at ages 11, 15, and 18 years in the 1993 Pelotas Birth Cohort.

|  | | **Men**^a^ | | | | | | | |  | | **Women**^a^ | | | | | | | | | |  |  |
| --- | --- | --- | --- | --- | --- | --- | --- | --- | --- | --- | --- | --- | --- | --- | --- | --- | --- | --- | --- | --- | --- | --- | --- |
|  | | Crude | | | |  | | Adjusted | |  | | Crude | | | |  | | Adjusted | | | |  |  |
|  | | OR (95% CI) | | p value^b^ | |  | | OR (95% CI) | p value^b^ |  | | OR (95% CI) | | p value^b^ | |  | | OR (95% CI) | | p value^b^ | |  |  |
| Non-violent crime | | | |  | |  | |  | |  | |  | |  | |  | |  | |  | |  | |
| Heart at 11 years | |  | | 0.043 | |  | |  | | 0.106 | |  | |  | | 0.499 | |  | |  | | 0.600 | |
| 4 (highest) | | 1.00 | |  | |  | | 1.00 | |  | |  | | 1.00 | |  | |  | | 1.00 | |  | |
| 3 | | 0.82 (0.55-1.21) | |  | |  | | 0.77 (0.52-1.16) | |  | |  | | 0.75 (0.42-1.35) | |  | |  | | 0.69 (0.38-1.25) | |  | |
| 2 | | 1.27 (0.88-1.85) | |  | |  | | 1.21 (0.82-1.77) | |  | |  | | 1.03 (0.60-1.76) | |  | |  | | 0.97 (0.56-1.68) | |  | |
| 1 (lowest) | | 1.31 (0.90-1.92) | |  | |  | | 1.21 (0.82-1.78) | |  | |  | | 1.09 (0.66-1.82) | |  | |  | | 1.04 (0.61-1.76) | |  | |
| Heart at 15 years | |  | | 0.022 | |  | |  | | 0.070 | |  | |  | | 0.506 | |  | |  | | 0.544 | |
| 4 (highest) | | 1.00 | |  | |  | | 1.00 | |  | |  | | 1.00 | |  | |  | | 1.00 | |  | |
| 3 | | 1.19 (0.77-1.82) | |  | |  | | 1.14 (0.74-1.76) | |  | |  | | 0.92 (0.51-1.68) | |  | |  | | 0.91 (0.49-1.68) | |  | |
| 2 | | 1.24 (0.84-1.85) | |  | |  | | 1.15 (0.77-1.73) | |  | |  | | 1.06 (0.60-1.86) | |  | |  | | 1.08 (0.60-1.94) | |  | |
| 1 (lowest) | | 1.57 (1.07-2.30) | |  | |  | | 1.45 (0.98-2.14) | |  | |  | | 1.17 (0.65-2.10) | |  | |  | | 1.15 (0.63-2.10) | |  | |
| Heart at 18 years | |  | | 0.007 | |  | |  | | 0.076 | |  | |  | | 0.049 | |  | |  | | 0.089 | |
| 4 (highest) | | 1.00 | |  | |  | | 1.00 | |  | |  | | 1.00 | |  | |  | | 1.00 | |  | |
| 3 | | 1.55 (1.04-2.30) | |  | |  | | 1.39 (0.93-2.09) | |  | |  | | 1.21 (0.65-2.27) | |  | |  | | 1.18 (0.62-2.23) | |  | |
| 2 | | 1.58 (1.08-2.31) | |  | |  | | 1.41 (0.95-2.08) | |  | |  | | 1.42 (0.80-2.53) | |  | |  | | 1.35 (0.75-2.45) | |  | |
| 1 (lowest) | | 1.79 (1.20-2.66) | |  | |  | | 1.50 (1.00-2.27) | |  | |  | | 1.74 (0.98-3.09) | |  | |  | | 1.63 (0.91-2.92) | |  | |

^a^ 2603 for men; 2645 for women

^b^ Wald test for linear trend

Covariates included in adjusted model: unplanned pregnancy, mother smoked in pregnancy, maternal alcohol use in pregnancy, maternal age, number of siblings, maternal education, and family income in perinatal period; child skin colour, smoking, drinking, physical activity, height, weight, blood pressure, maternal mental health at 11 years.

Table S4: Odds Ratio (OR) for violent and non-violent crime at age 18 according to quartiles of first and second heart rate measures taken at ages 18 years, in the 1993 Pelotas Birth Cohort.

|  | **Men**^a^ | | | | |  | **Women**^a^ | | | | |
| --- | --- | --- | --- | --- | --- | --- | --- | --- | --- | --- | --- |
|  | Crude | |  | Adjusted | |  | Crude | |  | Adjusted | |
|  | OR (95% CI) | p value^b^ |  | OR (95% CI) | p value^b^ |  | OR (95% CI) | p value^b^ |  | OR (95% CI) | p value^b^ |
| **Violent crime** | |  |  |  |  |  |  |  |  |  |  |
| 1^st^ heart rate measure |  | 0.005 |  |  | 0.123 |  |  | <0.001 |  |  | 0.002 |
| 4 (highest) | 1.00 |  |  | 1.00 |  |  | 1.00 |  |  | 1.00 |  |
| 3 | 1.30 (0.94-1.80) |  |  | 1.17 (0.82-1.67) |  |  | 1.72 (1.04-2.82) |  |  | 1.56 (0.90-2.71) |  |
| 2 | 1.56 (1.14-2.14) |  |  | 1.33 (0.95-1.88) |  |  | 1.48 (0.90-2.44) |  |  | 1.38 (0.80-2.38) |  |
| 1 (lowest) | 1.51 (1.11-2.05) |  |  | 1.28 (0.92-1.80) |  |  | 2.48 (1.56-3.96) |  |  | 2.34 (1.41-3.91) |  |
| 2^nd^ heart rate measure |  | 0.001 |  |  | 0.030 |  |  | <0.001 |  |  | 0.003 |
| 4 (highest) | 1.00 |  |  | 1.00 |  |  | 1.00 |  |  | 1.00 |  |
| 3 | 1.51 (1.09-2.09) |  |  | 1.46 (1.03-2.08) |  |  | 1.44 (0.88-2.35) |  |  | 1.45 (0.84-2.52) |  |
| 2 | 1.62 (1.18-2.21) |  |  | 1.48 (1.05-2.09) |  |  | 1.62 (1.00-2.63) |  |  | 1.61 (0.93-2.77) |  |
| 1 (lowest) | 1.74 (1.28-2.37) |  |  | 1.51 (1.07-2.11) |  |  | 2.16 (1.39-3.39) |  |  | 2.10 (1.27-3.46) |  |
| **Non violent crime** | |  |  |  |  |  |  |  |  |  |  |
| 1^st^ measure heart rate |  | 0.020 |  |  | 0.091* |  |  | 0.111 |  |  | 0.031 |
| 4 (highest) | 1.00 |  |  | 1.00 |  |  | 1.00 |  |  | 1.00 |  |
| 3 | 1.75 (1.16-2.65) |  |  | 1.65 (1.04-2.61) |  |  | 1.35 (0.71-2.55) |  |  | 1.49 (0.71-3.13) |  |
| 2 | 1.42 (0.94-2.16) |  |  | 1.22 (0.77-1.95) |  |  | 1.17 (0.62-2.23) |  |  | 1.36 (0.65-2.85) |  |
| 1 (lowest) | 1.79 (1.21-2.65) |  |  | 1.61 (1.04-2.51) |  |  | 1.71 (0.94-3.14) |  |  | 2.19 (1.10-4.39) |  |
| 2^nd^ measure heart rate |  | 0.020 |  |  | 0.180 |  |  | 0.076 |  |  | 0.031 |
| 4 (highest) | 1.00 |  |  | 1.00 |  |  | 1.00 |  |  | 1.00 |  |
| 3 | 1.39 (0.92-2.09) |  |  | 1.30 (0.82-2.04) |  |  | 0.86 (0.44-1.67) |  |  | 1.12 (0.53-2.36) |  |
| 2 | 1.60 (1.08-2.36) |  |  | 1.47 (0.95-2.27) |  |  | 1.26 (0.68-2.32) |  |  | 1.46 (0.71-2.97) |  |
| 1 (lowest) | 1.56 (1.06-2.30) |  |  | 1.34 (0.87-2.07) |  |  | 1.49 (0.85-2.62) |  |  | 1.90 (0.98-3.67) |  |

^a^ N for men between 1754 and 1541; N for women between 1772 and 1580, ^b^ Wald test for linear trend, * Wald test for heterogeneity
Covariates included in adjusted model: unplanned pregnancy, mother smoked in pregnancy, maternal alcohol use in pregnancy, maternal age, number of siblings, maternal education, and family income in perinatal period; child skin colour, smoking, drinking, physical activity, height, weight, blood pressure, maternal mental health at 11 years.
