# Supplementary material for: Low resting heart rate is associated with violence in late adolescence: a prospective birth cohort study in Brazil
Source: Int J Epidemiol. 2016 Jan 28;45(2):491–500. doi: 10.1093/ije/dyv340 (PMC4864875; doi:10.1093/ije/dyv340)
Supplement: Supplementary Data [file dyv340_supplementary_data.zip › Heart_rate_violence_Supplement_2015_12_14.pdf]

Table S1: Study measures by availability of crime data at age 18

	With crime data N = 3,618		Missing crime data N = 1,631
**Heart rate measures**			
Age 11 (bpm)		p=0.512	
Mean (SD)	78.5 (11.1)		78.2 (10.5)
Age 15 (bpm)		p=0.336	
Mean (SD)	81.9 (12.6)		81.4 (12.1)
Age 18 (bpm)		p<0.001	
Mean (SD)	73.6 (12.2)		76.2 (13.0)
**Perinatal measures**			
Unplanned pregnancy		p=0.571	
Yes	55.9%		57.0%
Mother smoked in pregnancy		p=0.233	
Yes	32.9%		34.5%
Mother used alcohol in pregnancy		p=0.580	
Yes	5.0%		5.3%
Maternal age (years)		p=0.444	
Mean (SD)	26.0 (6.4)		25.9 (6.4)
Number of pregnancies		p=0.123	
Mean (SD)	2.5 (1.7)		2.5 (1.8)
Maternal education (years)		p=0.168	
Mean (SD)	6.8 (3.5)		6.6 (3.7)
Family income (minimum wages)		p=0.827	
Mean (SD)	4.3 (5.9)		4.3 (5.8)
**Age 11 measures**			
Participant smoking		p=0.117	
Yes	3.5%		4.6%
Participant drinking alcohol		p=0.177	
Yes	17.1%		19.0%
Participant physical activity (mins/pw)		p=0.963	
Mean (SD)	414 (760)		416 (780)
Participant height (cm)		p=0.020	
Mean (SD)	1.46 (0.08)		1.46 (0.08)
Participant weight (kg)		p=0.051	
Mean (SD)	39.8 (10.2)		40.5 (11.0)
Participant systolic blood pressure		p=0.001	
Mean (SD)	101.5 (12.3)		103.0 (12.2)
Participant diastolic blood pressure		p=0.018	
Mean (SD)	63.2 (9.9)		64.0 (9.7)
Maternal mental health		p=0.359	
Mean (SD)	5.7 (4.5)		5.8 (4.5)

Notes. Column percents.

Table S2. Multiple imputation results: Odds Ratio (OR) for violent crime at age 18 according to heart rate quartiles at ages 11, 15, and 18 years in the 1993 Pelotas Birth Cohort.

	**Men[a]**				**Women[a]**			
	Crude		Adjusted		Crude		Adjusted	
	OR (95% CI)	p value[b]	OR (95% CI)	p value[b]	OR (95% CI)	p value[b]	OR (95% CI)	p value[b]
Violent crime								
Heart at 11 years		0.004		0.024		0.076		0.192
4 (highest)	1.00		1.00		1.00		1.00	
3	1.38 (1.00-1.90)		1.33 (0.96-1.86)		1.15 (0.75-1.76)		1.05 (0.68-1.62)	
2	1.45 (1.04-2.01)		1.40 (0.99-1.96)		1.22 (0.80-1.84)		1.14 (0.75-1.74)	
1 (lowest)	1.59 (1.16-2.17)		1.47 (1.06-2.02)		1.42 (0.96-2.10)		1.29 (0.86-1.94)	
Heart at 15 years		<0.001		0.003		0.318		0.659
4 (highest)	1.00		1.00		1.00		1.00	
3	1.47 (1.07-2.03)		1.43 (1.03-1.97)		1.19 (0.78-1.89)		1.15 (0.74-1.79)	
2	1.40 (1.01-1.95)		1.29 (0.92-1.81)		1.34 (0.89-2.01)		1.30 (0.86-1.99)	
1 (lowest)	1.88 (1.36-2.58)		1.75 (1.27-2.41)		1.22 (0.79-1.87)		1.08 (0.70-1.67)	
Heart at 18 years		0.001		0.012		0.001		0.012
4 (highest)	1.00		1.00		1.00		1.00	
3	1.42 (1.05-1.92)		1.32 (0.97-1.81)		1.37 (0.86-2.18)		1.31 (0.82-2.10)	
2	1.66 (1.24-2.24)		1.52 (1.12-2.06)		1.50 (0.96-2.36)		1.37 (0.87-2.16)	
1 (lowest)	1.69 (1.27-2.26)		1.46 (1.08-1.99)		1.97 (1.29-3.01)		1.72 (1.12-2.63)	

[a] 2603 for men; 2645 for women

[b] Wald test for linear trend

* Wald test for heterogeneity

Covariates included in adjusted model: unplanned pregnancy, mother smoked in pregnancy, maternal alcohol use in pregnancy, maternal age, number of siblings, maternal education, and family income in perinatal period; child skin colour, smoking, drinking, physical activity, height, weight, blood pressure, maternal mental health at 11 years.

Table S3. Multiple imputation results: Odds Ratio (OR) for non-violent crime at age 18 according to heart rate quartiles at ages 11, 15, and 18 years in the 1993 Pelotas Birth Cohort.

	**Men**[a]				**Women**[a]			
	Crude		Adjusted		Crude		Adjusted	
	OR (95% CI)	p value[b]	OR (95% CI)	p value[b]	OR (95% CI)	p value[b]	OR (95% CI)	p value[b]
Non-violent crime								
Heart at 11 years		0.043		0.106		0.499		0.600
4 (highest)	1.00		1.00		1.00		1.00	
3	0.82 (0.55-1.21)		0.77 (0.52-1.16)		0.75 (0.42-1.35)		0.69 (0.38-1.25)	
2	1.27 (0.88-1.85)		1.21 (0.82-1.77)		1.03 (0.60-1.76)		0.97 (0.56-1.68)	
1 (lowest)	1.31 (0.90-1.92)		1.21 (0.82-1.78)		1.09 (0.66-1.82)		1.04 (0.61-1.76)	
Heart at 15 years		0.022		0.070		0.506		0.544
4 (highest)	1.00		1.00		1.00		1.00	
3	1.19 (0.77-1.82)		1.14 (0.74-1.76)		0.92 (0.51-1.68)		0.91 (0.49-1.68)	
2	1.24 (0.84-1.85)		1.15 (0.77-1.73)		1.06 (0.60-1.86)		1.08 (0.60-1.94)	
1 (lowest)	1.57 (1.07-2.30)		1.45 (0.98-2.14)		1.17 (0.65-2.10)		1.15 (0.63-2.10)	
Heart at 18 years		0.007		0.076		0.049		0.089
4 (highest)	1.00		1.00		1.00		1.00	
3	1.55 (1.04-2.30)		1.39 (0.93-2.09)		1.21 (0.65-2.27)		1.18 (0.62-2.23)	
2	1.58 (1.08-2.31)		1.41 (0.95-2.08)		1.42 (0.80-2.53)		1.35 (0.75-2.45)	
1 (lowest)	1.79 (1.20-2.66)		1.50 (1.00-2.27)		1.74 (0.98-3.09)		1.63 (0.91-2.92)	

[a] 2603 for men; 2645 for women

[b] Wald test for linear trend

Covariates included in adjusted model: unplanned pregnancy, mother smoked in pregnancy, maternal alcohol use in pregnancy, maternal age, number of siblings, maternal education, and family income in perinatal period; child skin colour, smoking, drinking, physical activity, height, weight, blood pressure, maternal mental health at 11 years.

Table S4: Odds Ratio (OR) for violent and non-violent crime at age 18 according to quartiles of first and second heart rate measures taken at ages 18 years, in the 1993 Pelotas Birth Cohort.

	Men[a]				Women[a]			
	Crude		Adjusted		Crude		Adjusted	
	OR (95% CI)	p value[b]	OR (95% CI)	p value[b]	OR (95% CI)	p value[b]	OR (95% CI)	p value[b]
**Violent crime**								
1st heart rate measure		0.005		0.123		<0.001		0.002
4 (highest)	1.00		1.00		1.00		1.00	
3	1.30 (0.94-1.80)		1.17 (0.82-1.67)		1.72 (1.04-2.82)		1.56 (0.90-2.71)	
2	1.56 (1.14-2.14)		1.33 (0.95-1.88)		1.48 (0.90-2.44)		1.38 (0.80-2.38)	
1 (lowest)	1.51 (1.11-2.05)		1.28 (0.92-1.80)		2.48 (1.56-3.96)		2.34 (1.41-3.91)	
2nd heart rate measure		0.001		0.030		<0.001		0.003
4 (highest)	1.00		1.00		1.00		1.00	
3	1.51 (1.09-2.09)		1.46 (1.03-2.08)		1.44 (0.88-2.35)		1.45 (0.84-2.52)	
2	1.62 (1.18-2.21)		1.48 (1.05-2.09)		1.62 (1.00-2.63)		1.61 (0.93-2.77)	
1 (lowest)	1.74 (1.28-2.37)		1.51 (1.07-2.11)		2.16 (1.39-3.39)		2.10 (1.27-3.46)	
**Non violent crime**								
1st measure heart rate		0.020		0.091*		0.111		0.031
4 (highest)	1.00		1.00		1.00		1.00	
3	1.75 (1.16-2.65)		1.65 (1.04-2.61)		1.35 (0.71-2.55)		1.49 (0.71-3.13)	
2	1.42 (0.94-2.16)		1.22 (0.77-1.95)		1.17 (0.62-2.23)		1.36 (0.65-2.85)	
1 (lowest)	1.79 (1.21-2.65)		1.61 (1.04-2.51)		1.71 (0.94-3.14)		2.19 (1.10-4.39)	
2nd measure heart rate		0.020		0.180		0.076		0.031
4 (highest)	1.00		1.00		1.00		1.00	
3	1.39 (0.92-2.09)		1.30 (0.82-2.04)		0.86 (0.44-1.67)		1.12 (0.53-2.36)	
2	1.60 (1.08-2.36)		1.47 (0.95-2.27)		1.26 (0.68-2.32)		1.46 (0.71-2.97)	
1 (lowest)	1.56 (1.06-2.30)		1.34 (0.87-2.07)		1.49 (0.85-2.62)		1.90 (0.98-3.67)	

[a] N for men between 1754 and 1541; N for women between 1772 and 1580, [b] Wald test for linear trend, * Wald test for heterogeneity
Covariates included in adjusted model: unplanned pregnancy, mother smoked in pregnancy, maternal alcohol use in pregnancy, maternal age, number of siblings, maternal education, and family income in perinatal period; child skin colour, smoking, drinking, physical activity, height, weight, blood pressure, maternal mental health at 11 years.
